# Band Gap Control in Bilayer Graphene by Co-Doping with B-N Pairs

**DOI:** 10.1038/s41598-018-35671-2

**Published:** 2018-12-06

**Authors:** M. Alattas, U. Schwingenschlögl

**Affiliations:** King Abdullah University of Science and Technology (KAUST), Physical Science and Engineering Division (PSE), Thuwal, 23955-6900 Saudi Arabia

## Abstract

The electronic band structure of bilayer graphene is studied systematically in the presence of substitutional B and/or N doping, using density functional theory with van der Waals correction. We show that introduction of B-N pairs into bilayer graphene can be used to create a substantial band gap, stable against thermal fluctuations at room temperature, but otherwise leaves the electronic band structure in the vicinity of the Fermi energy largely unaffected. Introduction of B-N pairs into B and/or N doped bilayer graphene likewise hardly modifies the band dispersions. In semiconducting systems (same amount of B and N dopants), however, the size of the band gap is effectively tuned in the presence of B-N pairs.

## Introduction

Absence of a band gap limits applications of graphene in electronics, calling for band gap engineering^1,2^. Substitutional doping with foreign atoms is one of the main approaches to intrinsically alter the electronic properties^3–5^. In particular, graphene is often doped with B^6^ and N^7^, as these atoms have radii (B: 87 pm; N: 56 pm) similar to that of C (67 pm) and the planar geometry therefore is preserved^8,9^. Gradual transition from n-type to p-type character in contact with air can be neutralized by intentional overdoping^10–15^. Bilayer graphene early has been proposed as viable alternative to the monolayer^16^. In the case of AA stacking the material consists of two graphene layers located directly on top of each other, whereas in the case of AB stacking half of the C atoms from one layer sits on top of C atoms of the other layer and the other half sits on top of hollow sites. Both stackings have been observed experimentally^17,18^, AB stacking being 5 meV per C atom lower in energy than AA stacking^19^ and nowadays can be grown in high quality and with large area^20^. It has been demonstrated that both B and N doping requires less energy than in the monolayer^21^.

Introduction of one B or one N atom into AB stacked bilayer graphene has been studied in ref.^22^ both for the top and hollow sites (which refers here and in the following to the position of the dopant atom relative to the other layer) and similar electronic band structures have been found for the two sites in each case. B and N favor hollow sites over top sites by 11 meV and 16 meV, respectively^21^. In AB stacked bilayer graphene with one N atom in each layer the total energy declines significantly for increasing distance between the two dopant atoms, while for corresponding doping with B atoms hardly any dependence of the total energy on the distance is found^23^. Codoping of B and N in graphene (monolayer) is energetically favorable in the form of B-N pairs in ortho position, for example, by 20 meV as compared to next nearest neighbours^24^. Reference^25^ has studied bilayer graphene with one B atom in one layer and one N atom in the other, claiming that the electronic properties depend on the occupied sublattice rather than on the mutual distance. In the present work, we investigate the electronic properties of bilayer graphene in the more general case that B and N also can be codoped in the same layer. Interestingly, it turns out that the presence of such B-N pairs has only minor effects on the electronic band structure except for the position of the band edges in the case that the system is semiconducting. For this reason, our results indicate that intentional doping of B-N pairs is an effective way to control the size of the band gap.

## Computational Methods

Spin-polarized first-principles calculations are performed using the Vienna ab-initio simulation package^26^ and the generalized gradient approximation for the exchange-correlation potential (Perdew-Burke-Ernzerhof parameterization), taking into account a van der Waals correction with Becke-Johnson damping^27^. The plane wave energy cutoff is set to 500 eV. We employ a Γ-centered 2 × 2 × 1 k-mesh for the structure optimization and a Γ-centered 8 × 8 × 1 k-mesh for evaluating the electronic properties. Both for AA and AB stacking, a 5 × 5 supercell of bilayer graphene is constructed with a total of 100 C atoms (to consider doping effects) to which a 16 Å thick vacuum layer is attached along the *c*-axis in order to avoid artificial interaction between periodic images. The interlayer distance after structure optimization turns out to be 3.6 Å for AA stacking and 3.4 Å for AB stacking, in agreement with the experimental observations in refs^28,29^. These values are not modified by the presence of dopant atoms for the concentrations considered in this study. We obtain for both stackings an in-plane lattice parameter of 2.46 Å and a C-C bond length of 1.42 Å. As in no case atoms develop significant magnetic moments (≤0.01 *μ*_*B*_), we present in the following spin-degenerate band structures.

## Results and Discussion

**Figure 1:**
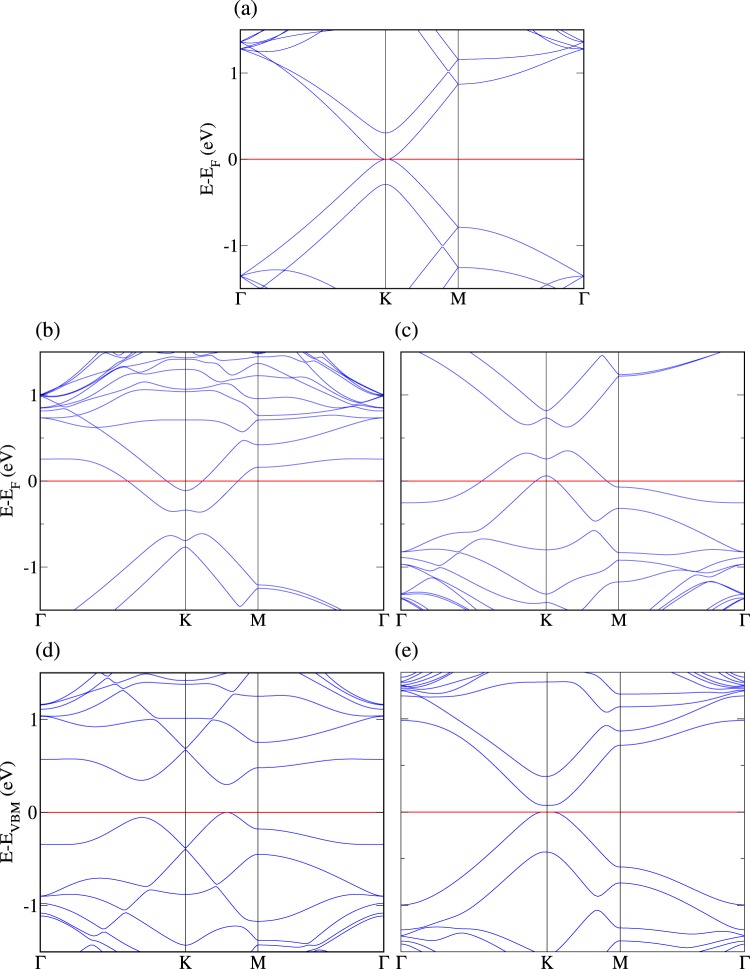
Electronic band structure of AB stacked bilayer graphene: (**a**) undoped, (**b**) doped with one N atom in one layer, (**c**) doped with one B atom in one layer, (**d**) doped with one N atom in one layer and one B atom in the other, and (**e**) doped with one B-N pair in one layer.

For reference, we show the electronic band structure of AB stacked bilayer graphene in Fig. 1(a) in the undoped case, in Fig. 1(b) doped with one N atom in one layer, and in Fig. 1(c) doped with one B atom in one layer. All these results agree with the existing literature^22,30^. We next introduce one B atom into one layer and one N atom into the other, representing a dopant concentration of 2%, and examine different distances between them as well as doping at both the top and hollow sites. In each case we obtain minimal energy for minimal distance between the dopant atoms. The energetically favorable configuration with AB stacking has 467 meV (~5 meV per C atom) lower energy than that with AA stacking. In the case of AB stacking, when B sits at a top site and N at a hollow site the energy is 14 meV lower than when both sit at hollow sites, 75 meV lower than when N sits at a top site and B at a hollow site, and 65 meV lower than when both sit at top sites. The electronic band structure of the minimum energy configuration, see Fig. 1(d), agrees with the result of ref.^25^. There are two buried Dirac cones at the K point, which can be attributed to the layers doped with B (unoccupied cone) and N (occupied cone), and we obtain a band gap of 298 meV.

Turning to the situation that both the B and N atoms are located in the same layer while the other layer is undoped, we confirm that for both AA and AB stacking the formation of a B-N pair is energetically favorable, as a strong covalent bond is formed^31^. Concerning the position of the B-N pair relative to the other layer we find that N occupying the hollow site gives an energy advantage of 22 meV, which agrees with the finding of ref.^21^ that N favors the hollow site in bilayer graphene more strongly than B (by 22 meV). It turns out that the total energy is 590 meV (~6 meV per C atom) lower for AB stacking than for AA stacking. The electronic band structure of the minimum energy configuration is shown in Fig. 1(e). We observe parabolic bands in the vicinity of the Fermi energy, as expected for AB stacked bilayer graphene (see Fig. 1(a) and ref.^32^), with a band gap of 70 meV due to the presence of the B-N pair. As a remark, we note that the electronic properties depend on the sites (top or hollow; characterized by different coordination geometries) occupied by the dopant atoms rather than on the occupied sublattice as claimed in ref.^25^.

**Figure 2:**
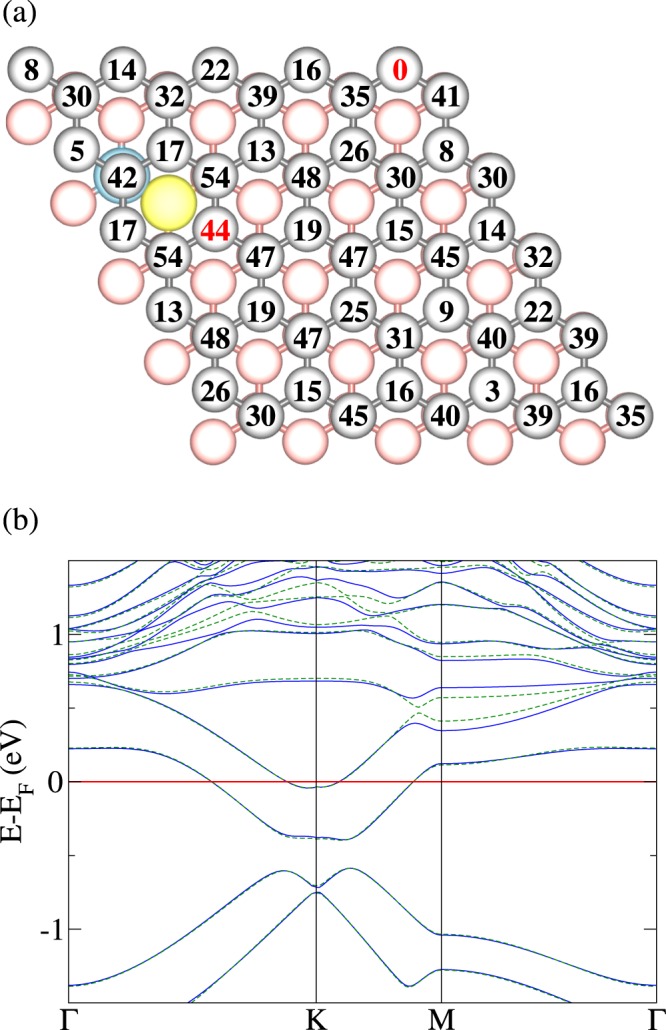
(**a**) Top view of AB stacked bilayer graphene with one B-N pair in the bottom layer (B blue, N yellow, top layer gray, bottom layer red). The numbers represent the total energy in meV for location of an additional N atom at the respective site of the other (top) layer. (**b**) Electronic band structures for location of the N atom at the sites indicated in (**a**) by red numbers: next to the B-N pair (blue solid line) and far from the B-N pair (green dashed line).

We next study, for different relative positions, one B-N pair in one layer and one N atom in the other. It turns out that the N atom of the B-N pair still favors the hollow site, as the coordination geometry is not modified. The numbers in Fig. 2(a) are total energy differences in meV for location of the N atom at the respective site relative to the minimum energy location. We observe significant variations in the total energy. Comparing the electronic band structures obtained for the highest and lowest energy configurations (at distances of 2.13 Å and 6.41 Å, respectively), however, shows close similarity, see Fig. 2(b). We observe a sizeable shift of all bands to lower energy, with respect to Fig. 1(e), reflecting an n-type character due to the extra charge of the N atom. On the other hand, the band dispersions in the vicinity of the Fermi energy closely resemble those of Fig. 1(b). The presence of a B-N pair in one layer increases the binding energy to the other layer by 37 meV due to interaction between the B-N dipole and the induced dipole in the other layer, showing that B-N pairs can be used to enhance the interlayer coupling.

**Figure 3:**
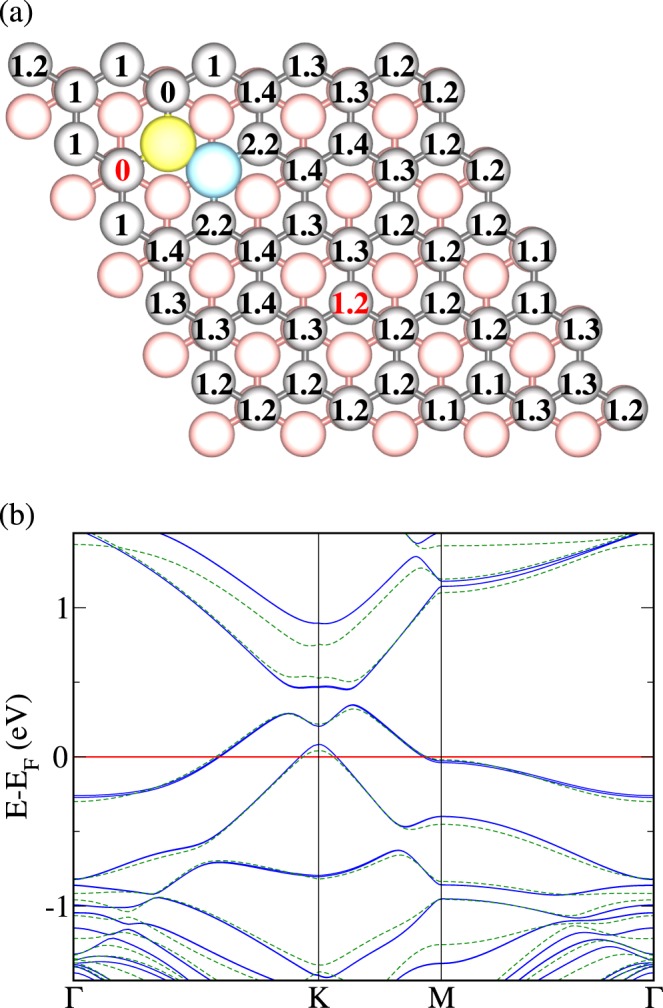
(**a**) Top view of AB stacked bilayer graphene with one B-N pair in the top layer (B blue, N yellow, top layer gray, bottom layer red). The numbers represent the total energy in eV for location of an additional B atom at the respective site of the same (top) layer. (**b**) Electronic band structures for location of the B atom at the sites indicated in (**a**) by red numbers: next to the B-N pair (blue solid line) and far from the B-N pair (green dashed line).

Keeping one layer undoped, we now add one B atom to the layer containing the B-N pair. The numbers in Fig. 3(a) are the total energy differences in eV for location of the additional B atom at the respective site relative to the minimum energy location. We observe a strong tendency to bond to the N atom of the B-N pair (forming a B-N-B triple by occupying a top site), as the local charge deficit can be partially compensated by the excess charge at the N site. Despite large differences in the total energy, Fig. 3(b) demonstrates that the electronic band structures are similar for different positions of the additional B atom. We also observe that the band dispersions in the vicinity of the Fermi energy are closely related to those of Fig. 1(c).

**Figure 4:**
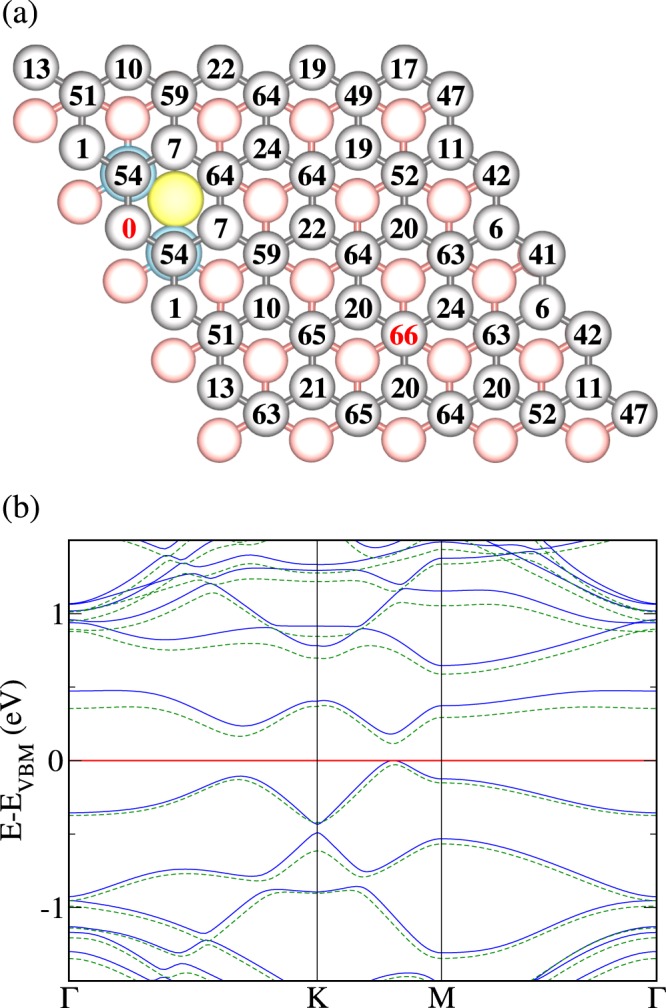
(**a**) Top view of AB stacked bilayer graphene with one B-N-B triple in the bottom layer (B blue, N yellow, top layer gray, bottom layer red). The numbers represent the total energy in meV for location of an additional N atom at the respective site of the other (top) layer. (**b**) Electronic band structures for location of the N atom at the sites indicated in (a) by red numbers: next to the B-N-B triple (blue solid line) and far from the B-N-B triple (green dashed line).

We finally study, for different relative positions, one N atom in one layer and one B-N-B triple in the other. The numbers in Fig. 4(a) are the total energy differences in meV for location of the N atom at the respective site relative to the minimum energy location. Similar to the situation of Fig. 2, the N atom favors hollow over top sites. We compare in Fig. 4(b) the electronic band structures obtained for the highest (N far from the B-N-B triple) and lowest (N next to the two B atoms of the B-N-B triple) energy configurations, finding close similarity, however, with a difference of 43 meV in the size of the band gap. The band dispersions in the vicinity of the Fermi energy resemble those of Fig. 1(d), while the band gap is much smaller (148 meV instead of 298 meV for the lowest energy configuration, for example). The fact that the presence of B-N pairs in bilayer graphene hardly affects the shape of the electronic bands but strongly alters the size of the band gap makes it possible to tune these quantities independently of each other.

In conclusion, the effects of B and/or N doping on bilayer graphene have been studied by first-principles calculations based on density functional theory. Special attention has been given to the modifications of the electronic band structure due to the presence of B-N pairs. We have demonstrated that such B-N pairs hardly modify the band dispersions in bilayer graphene, while opening a substantial band gap (of 70 meV in the case of 2% dopant concentration, for example). Similarly, introduction of B-N pairs into N and/or B doped bilayer graphene largely maintains the electronic band structure (for any relative position between the dopant species). Interestingly, for semiconducting systems (same amount of B and N dopants) our results show that the presence of B-N pairs shifts the band edges (almost rigidly) towards each other, i.e., the band gap shrinks. For this reason, introduction of B-N pairs into bilayer graphene can be used to tune the size of the band gap without critically affecting the band dispersions.
